# Characteristic Factors of Aspiration Pneumonia to Distinguish from Community-Acquired Pneumonia among Oldest-Old Patients in Primary-Care Settings of Japan

**DOI:** 10.3390/geriatrics5030042

**Published:** 2020-07-07

**Authors:** Toshie Manabe, Kazuhiko Kotani, Hiroyuki Teraura, Kensuke Minami, Takahide Kohro, Masami Matsumura

**Affiliations:** 1 Division of Community and Family Medicine, Center of Community Medicine, Jichi Medical University, Shimotsuke-City, Tochigi 329-0498, Japan; manabe@jichi.ac.jp (T.M.); m05062ht@jichi.ac.jp (H.T.); 2 Division of Infectious Diseases, Jichi Medical University Hospital, Shimotsuke-City, Tochigi 329-0498, Japan; kens37mi@jichi.ac.jp; 3 Data Science Center, Jichi Medical University, Shimotsuke-City, Tochigi 329-0498, Japan; takahide.kohro@jichi.ac.jp; 4 Division of General Medicine, Center of Community Medicine, Jichi Medical University, Shimotsuke-City, Tochigi 329-0498, Japan; nephron@jichi.ac.jp

**Keywords:** aspiration pneumonia, community-acquired pneumonia, primary care, dysphagia, nursing home

## Abstract

**Background:** Aspiration pneumonia (AsP), a phenotype of community-acquired pneumonia (CAP), is a common and problematic disease with symptomless recurrence and fatality in old adults. Characteristic factors for distinguishing AsP from CAP need to be determined to manage AsP. No such factorial markers in oldest-old adults, who are often seen in the primary-care settings, have yet been established. **Methods:** From the database of our Primary Care and General Practice Study, including the general backgrounds, clinical conditions and laboratory findings collected by primary care physicians and general practitioners, the records of 130 patients diagnosed with either AsP (n = 72) or CAP (n = 58) were extracted. Characteristic factors associated with the diagnosis of AsP were statistically compared between AsP and CAP. **Results:** The patients were older in the AsP group (median 90 years old) than in the CAP group (86 years old). The body temperature, heart rate, and diastolic blood pressure were lower in the patients with AsP than in those with CAP. Witnessed meal dysphagia by families and caregivers was reported only in AsP. Living in a nursing home, comorbidities of cerebral infarction and dementia (as positive factors) and hypertension (as a negative factor) were considered predictive to diagnose AsP in a stepwise logistic regression analysis. **Conclusions:** Among oldest-old adults in primary-care settings, living in a nursing home and the dysphagia risks are suggested to be characteristic factors for diagnosing AsP. Age and some relevant clinical information may help manage AsP and also be useful for families and caregivers.

## 1. Introduction

Aspiration pneumonia (AsP) is a lung infection induced by pathogens and materials from the mouth and stomach, and the risk is generally high in older adults [1]. The development of AsP is a socio-clinical burden, and identifying and understanding AsP is getting crucial in rapidly aging societies, like Japan [1,2,3,4].

AsP is recognized as a phenotype of community-acquired pneumonia (CAP), and several studies have indicated that 7–24% of cases of CAP are due to aspiration [5,6]. A Japanese multicenter prospective study reported that approximately 60% of hospitalized patients with CAP were diagnosed with AsP [7]. AsP is more severe than non-aspiration pneumonia [4,6], with higher rates of mortality and recurrence as well as increased lengths of hospital stay compared with CAP [3,6,8]. In some cases, AsP can prompt the development of necrotizing pneumonia and/or lung abscess, leading to a prolonged course of antibiotics and surgery [9]. These findings underscore the importance of managing AsP compared with CAP. 

The diagnosis of pneumonia is generally troublesome, especially in older adults [10]. The standard criteria for a diagnosis of pneumonia are the presence of acute respiratory symptoms and fever associated with newly identified and modified infiltrates on chest radiography [11,12]. Among older adults, however, the clinical symptoms and signs of pneumonia are atypical, and chest radiography abnormalities can be non-specific [13,14,15,16]. To make the diagnostic criteria of AsP and the preventative measures to stop its recurrence is an issue among older adults [17]. 

In the primary-care settings, oldest-old patients with AsP are often seen by primary care physicians and general practitioners [18]. Pneumonia, including AsP, appears in the long-term care as provided in nursing homes, and the patients also visit medical facilities, which is an issue for caregivers [19,20]. Clinically diagnostic markers to determine AsP in such settings have not yet to be elucidated; therefore, the present study aimed to clarify the characteristic factors for distinguishing AsP from CAP in primary-care settings in Japan. 

## 2. Materials and Methods

### 2.1. Study Design, Setting, and Patients

An observational study was conducted using our database from the Primary Care and General Practice Study (PCGP study), which was approved by the Institutional Review Board of Jichi Medical University (Approval No. CL 18–150). The PCGP study comprises 20 medical facilities (community-based hospitals and clinics) across Japan (Supplemental Table S1), and its database was compiled by the study’s investigators based on a clinical record/chart review conducted at their practice sites for adult patients with common diseases, including pneumonia, between April 2013 and March 2019. The data of the PCGP study were collected and managed using the Research Electronic Data Capture (REDCap) electric data capture tools hosted at Jichi Medical University [21,22]. The REDCap is a secure, web-based software platform designed to support the data capture for research, providing an intuitive interface for validated data capture; audit trails for tracking data manipulation and export procedures; automated export procedures for seamless data downloads to common statistical packages; and procedures for data integration and interoperability with external sources [21,22]. Our database consisted of the clinical process-related data for the diagnosis of each patient (i.e., demographics, clinical conditions (such as chief complaints, medical history, and physical manifestations), and laboratory findings). The present study extracted these data from patients with AsP and CAP and then analyzed the characteristic factors for the patients. 

The investigators (primary care physicians and general practitioners) diagnosed AsP based on each patient’s overall clinical assessment, risk factors for aspiration, and/or chest radiograph abnormalities [12]. Specialized examinations, such as the swallowing function, were not always available for evaluating AsP in the practice sites. Using the database of eligible patients, the general backgrounds, clinical conditions, and laboratory findings were analyzed. Most patients visited the study site (in an emergency or ambulatory care room). Laboratory blood tests were generally obtained during hospitalization (thus, not all patients had laboratory findings). 

### 2.2. Statistical Analyses

Descriptive statistics were presented as proportions for categorical variables and the mean (standard deviation (SD]) or median (interquartile range (IQR)) for continuous variables. Comparisons between the AsP and CAP patient groups were assessed using the chi-squared test or Fisher’s exact test for categorical variables and Student’s *t*-test or the Mann–Whitney U test for continuous variables, according to the data distribution (using the Shapiro–Wilk test). 

Factors associated with the diagnosis of AsP were also estimated by a logistic regression analysis with independent variables (age, sex, place to live as nursing homes [19,20], main complaints, comorbidities, psychical manifestations (these variables were recorded in all patients and thought to be useful for the diagnosis)) if *p* was <0.05 and the odds ratio (OR) was >2.0 according to a univariate analysis in the model. A stepwise selection was used to select variables, which were confirmed by the forced entry method.

Data were analyzed using the IBM SPSS software program, version 25.0 (IBM Corp., Armonk, NY, USA). For all of the analyses, significance levels were two-tailed, and *p* < 0.05 was considered to indicate significance.

## 3. Results

### 3.1. General Backgrounds of Patients with AsP and CAP

Of the 130 total patients, 72 (55.4%) were diagnosed with AsP, and 58 (44.6%) were diagnosed with CAP at the time of data extraction. Table 1 shows the general background data of the patients with AsP and CAP.

The median age of patients with AsP was 90 (IQR, 81–93) years old, which was higher than that in the patients with CAP (86 (IQR, 76–90) years old). There were no marked differences in sex distribution between patients with AsP and CAP. Approximately 67% of patients with AsP were residents in a nursing home (including a geriatric facility), with a high OR (3.889; 95% confidence interval (CI), 1.419–10.660) compared to the patients with CAP. In terms of comorbidities, patients with AsP showed a higher prevalence of dementia (OR, 3.267; 95% CI, 1.388–7.689) and cerebral infarction (OR, 3.052; 95% CI, 1.362–6.837) than those with CAP. Other comorbidities showed no significant differences between the groups. The risk of experiencing fracture tended to be high among patients with AsP (OR, 3.667; 95% CI, 0.982–13.685) compared to those with CAP. Choking easily was reported in 20.8% of patients with AsP (OR, 3.553; 95% CI, 1.109–11.379). Only patients with AsP had a gastrostomy.

### 3.2. Clinical Conditions of Patients with AsP and CAP

Table 2 shows the clinical conditions (collected at the first visit to the study site) of patients with AsP and CAP. Approximately 70% of patients with AsP and 90% of those with CAP visited an emergency room, either by ambulance or at a time outside of the clinic’s office hours. High proportions of patients with AsP (40.3%) and CAP (37.9%) visited the study sites on the same day as the onset.

The most frequent complaint was fever in both patients with AsP (75.0%) and those with CAP (63.8%); however, there were no significant differences between the groups among the complaints, except for a low SpO_2_ in patients with AsP. Witnessed aspiration during meals was reported in 23.6% of patients with AsP but in 0% of those with CAP. Regarding patients’ physical manifestations, body temperature, heart rate, and diastolic blood pressure were significantly lower in patients with AsP than in those with CAP.

### 3.3. Laboratory Findings of Patients with AsP and CAP

Laboratory findings of the first blood tests are shown in Table 3, although the laboratory findings were reported in only a limited number of patients. No factors showed significant differences between the groups.

### 3.4. Characteristic Factors of AsP Using a Logistic Regression Analysis

When factors related to the diagnosis of AsP were estimated (Table 4), living in a nursing home, cerebral infarction, and dementia were positive factors of the diagnosis of AsP. Hypertension showed a negative association with the diagnosis of AsP.

## 4. Discussion

The present study examining oldest-old adults with pneumonia in the primary-care settings revealed that living in a nursing home and having comorbidities with risks of dysphagia, such as cerebral infarction and dementia, were preferentially characteristic factors that could distinguish AsP from CAP. Hypertension had a negative association with the diagnosis of AsP. As there have been few studies of clinical markers to determine AsP for oldest-old adults, the findings will aid in the management of AsP in such adults with suspected pneumonia.

Living in a nursing home can be a situation that is associated with the development of AsP [2,4,5,6,7,19,20,23], as such homes provide nursing care for geriatric syndrome and other disabilities. It is also important to note that pneumonia among patients who live in a nursing home is considered nursing- and healthcare-associated pneumonia (NHCAP), mainly being caused by drug-resistant bacteria [24,25]. Patients with NHCAP are typically older and show a poorer prognosis than those with CAP [26]. In the present study, the data of bacteriological tests, including the assessment of drug-resistant pathogens, were unavailable, so an etiological approach will be necessary to prevent recurrent pneumonia, such as AsP [27].

In the present study, dementia and cerebral infarction were found to be factors associated with AsP. Risk factors for aspiration are recognized to include an impaired consciousness, weakness, and swallowing dysfunction [2,23]. Dysphagia frequently occurs as the result of cerebral vascular disease and/or degenerative diseases of the cerebral nervous system, including dementia [28]. A study reported that patients with silent cerebral infarction had a higher risk of developing pneumonia than those with normal findings of head computed tomography [29]. A study examining patients living in a geriatric facility also suggested dementia as a risk factor for the development of AsP [30]. These can partly support the results of the present study.

To narrow down the characteristic factors among many variables with potential collinearity, a stepwise logistic regression analysis was used to extract the variables in the present study. The selection of the variables was reasonable in the methodology; however, this method could have been too strict, and real-world unselected information might also contribute to clinical practice. Therefore, some clinical information characterized by comparative statistics (i.e., an older age and low body temperature, heart rate, and diastolic blood pressure, as well as witnessed meal dysphagia) may aid in the management of AsP and help families and caregivers prevent AsP.

AsP involves misdirection of the oropharyngeal or gastric contents into the larynx and lower respiratory tract, and the aspiration of oropharyngeal secretions increases with age [2,4,5,6,7,23]. When primary care physicians and general practitioners vaguely think that AsP develops equally (regardless of age) among older adults, it may be of value to suggest that the risk of AsP can still increase with age even among oldest-old adults.

On the other hand, physical functions are seemingly relevant indicators related to aging rather than chronological age among older people [31]. Regarding the physical manifestations, the body temperature, heart rate, and diastolic blood pressure in patients with AsP could be lower than in patients with CAP. It is usable to focus attention on these manifestations for AsP, as primary care physicians and general practitioners tend to rely on the physical manifestations in daily practice. A fever is often absent in older adults with pneumonia; consequently, the heart rate does not increase [9,32]. In previous studies, the systolic heart function did not decrease with age [33], whereas the diastolic ability did decrease [34,35]. A low blood pressure is used as a marker in CURB-65 and A-DROP, which are the pneumonia severity indexes recommended by the British Thoracic Society [36] and the Japanese Respiratory Society [12], respectively. Lowering blood pressure as late-life physical dynamics indicates a potentially poor health with comorbidities [37,38]. This earlier knowledge may be associated with our present findings that hypertension was a negative factor of AsP in the logistic regression analysis and a low diastolic blood pressure appeared in AsP. The physical manifestations are thus indicative to be carefully evaluated in order to aptly manage AsP. Aging-prone pathophysiology more observed in older adults with AsP than those with CAP may merit further research. A comparison of the physical manifestations in those with AsP to healthy older people might be needed.

Aspiration is generally not witnessed, and even healthy people passively aspirate oropharyngeal secretions while asleep at night [39,40]. However, in the present study, witnessed meal dysphagia was reported by family members and caregivers in patients with AsP, whereas meal dysphagia was not reported for any patients with CAP. Meal dysphagia is influenced by a decreased food bolus-forming ability [41]. Insufficient food-bolus formation and chewing owing to missing teeth, as well as deterioration in tongue movements, increase the risk of aspiration [41,42,43]. In Japan, older adults who live in nursing homes receive 24-h supervision by caregivers. Particularly during mealtimes, caregivers carefully watch older adults or help them to eat. On the other hand, community-dwelling older adults living at home may eat meals alone during the daytime more often than residents in nursing homes. Paying close attention to older adults during meals is suggested to be useful for the prevention and early detection of meal aspiration leading to AsP.

Several limitations associated with the present study warrant mention. This study used the comparative design by cases extracted from the dataset. While the set was made using real-world data, the study might include information bias, as the preconception by study investigators when the diagnosis was not blinded. The variables of the database were not always similarly formatted for all patients and those with pneumonia. For example, although having gastro-esophageal reflux, a risk factor of AsP, was not detected in the study, this might simply have been due—at least in part—to the unformatted collection of data. Furthermore, the laboratory, bacteriological, and chest radiographic findings were not always recorded in all patients. Indeed, even though chest radiography was not performed in the study sites of the primary-care physicians and general practitioners who first diagnosed the patients, the diagnosis of pneumonia was confirmed in referred/hospitalized hospitals using radiography in all patients with AsP and CAP, following the guidelines [12]. However, a previous study indicated that the detection failure rate of chest radiographs was approximately 30% among patients with NHCAP [44], underscoring the importance of considering patients’ overall characteristics in the diagnosis of pneumonia. Given these limitations, further investigations with a well-formatted prospective design will be required to confirm the present findings.

In conclusion, among oldest-old adults in the primary-care setting, it is relevant to know their living situation (e.g., in a nursing home) and the presence of underlying diseases with risks of dysphagia (e.g., cerebral infarction and dementia) to diagnose AsP. Some clinical information (i.e., an older age and low body temperature, heart rate, and diastolic blood pressure, as well as witnessed meal dysphagia) may also aid in the management of AsP and help families and caregivers prevent AsP. As societies around the world age, these findings should be further investigated to aid in the management of pneumonia among oldest-old adults in the primary-care settings.

## Figures and Tables

**Table 1:** General background data of patients with AsP and CAP.

	AsP	CAP	*p* Value	OR	95% CI
	n = 72	n = 58			
Age, years, median (IQR)	90 (81–93)	86 (76–90)	0.027		
<65	2 (2.8)	7 (12.1)	0.062		
65–74	7 (9.7)	6 (10.3)			
75–89	26 (36.1)	26 (44.8)			
≥90	37 (51.4)	19 (32.8)			
Male, n (%)	34 (47.2)	30 (51.7)	0.724	0.835	0.418–1.669
Residence, n (%) (n = 76)					
Nursing home (including geriatric facility)	35 (68.6)	9 (36.0)	0.013	3.889	1.419–10.660
Home	16 (31.4)	16 (64.0)			
Comorbidity, n (%)					
Dementia	27 (37.5)	9 (15.5)	0.006	3.267	1.388–7.689
Cerebral infarction	30 (41.7)	11 (19.0)	0.008	3.052	1.362–6.837
Heat failure	3 (4.2)	7 (12.1)	0.109	0.317	0.078–1.285
Angina/arrhythmia	3 (4.2)	3 (5.2)	1.000	0.797	0.155–4.105
Hypertension	12 (16.7)	16 (27.6)	0.141	0.525	0.225–1.223
Diabetes mellitus	3 (4.2)	8 (13.8)	0.062	0.272	0.069–1.076
Pneumonia history	11 (15.3)	5 (8.6)	0.293	1.911	0.624–5.855
Asthma	4 (5.6)	5 (8.6)	0.511	0.624	0.160–2.437
Malignant neoplasm	8 (11.1)	6 (10.3)	1.000	1.083	0.353–3.320
Insomnia	5 (6.9)	0 (0.0)	0.065	-	-
Fracture	12 (16.7)	3 (5.2)	0.054	3.667	0.982–13.685
Daily states					
Choking easily	15 (20.8)	4 (6.9)	0.044	3.553	1.109–11.379
Gastrostomy	6 (8.3)	0 (0.0)	0.033	-	-

AsP, aspiration pneumonia; CAP, community-acquired pneumonia; OR, odds ratio; CI, confidence interval; IQR, interquartile range.

**Table 2:** Clinical conditions, taken at the first visit to the study site, in patients with AsP and CAP.

	AsP n = 72	CAP n = 58	*p* Value
Type of visits to study sites, n (%) (n = 84)			0.057
Emergency	38 (69.1)	26 (89.7)	
Ambulatory care	17 (30.9)	3 (10.3)	
Time until visiting the study site from the onset, n (%) (n = 116)			0.857
Same day as the onset	29 (40.3)	22 (37.9)	
1 day	15 (20.8)	13 (22.4)	
2–3 days	10 (13.9)	13 (22.4)	
≥4 days	6 (10.0)	8 (14.3)	
Main complaints, n (%)			
Dyspnea	11 (15.3)	7 (12.1)	0.622
Fever	54 (75.0)	37 (63.8)	0.182
Vomiting	3 (4.2)	1 (1.7)	0.628
Sputum	12 (16.7)	14 (24.1)	0.378
Consciousness disturbance	6 (8.3)	5 (8.6)	1.000
Anorexia	7 (9.7)	4 (6.9)	0.754
Wheezing	3 (4.2)	1 (1.7)	0.628
Low SpO_2_	6 (8.3)	0 (0.0)	0.033
Others	5 (6.9)	5 (8.6)	0.751
Other complaints, n (%)			
Aspiration during meals	17 (23.6)	0 (0.0)	<0.001
Physical manifestations, n (%)			
Body temperature (n = 108)	37.5 (0.9)	37.9 (0.9)	0.033
Heart rate (n = 81)	87.0 (24.8)	99.4 (24.1)	0.028
Respiration rate (n = 66)	25.0 (12.1)	26.1 (10.3)	0.719
Systolic blood pressure (n = 102)	124.9 (24.3)	130.3 (26.0)	0.276
Diastolic blood pressure (n = 102)	70.0 (15.4)	77.8 (18.1)	0.020
SpO_2_ (n = 79)	90.2 (8.7)	90.2 (6.3)	0.994

AsP, aspiration pneumonia; CAP, community-acquired pneumonia; SpO_2_, Oxygen saturation.

**Table 3:** Laboratory findings (available in limited patients) of the first blood tests in patients with AsP and CAP.

Parameters, Mean (SD)	AsP n = 72	CAP n = 58	*p* Value
WBC, count/µL (n = 70)	12,212.5 (6802.3)	11,983 (4901.0)	0.876
CRP, mg/dL (n = 70)	8.1 (7.6)	11.5 (9.7)	0.111
AST, U/L (n = 42)	28 (17.2)	55.8 (126.0)	0.299
ALT, U/L (n = 42)	21 (19.1)	26.5 (28.8)	0.438
LD, mg/dL (n = 31)	222.6 (66.7)	250 (137.7)	0.487
BUN, mg/dL (n = 51)	25 (14.6)	26.6 (18.1)	0.735
Creatinine, mg/dL (n = 52)	1.2 (2.2)	1.4 (2.0)	0.795

AsP, aspiration pneumonia; CAP, community-acquired pneumonia; SD, standard deviation. WBC, white blood cell; CRP, C-reactive protein; AST, aspartate aminotransferase; ALT, alanine aminotransferase; LD, lactate dehydrogenase; BUN, blood urea nitrogen.

**Table 4:** Characteristic factors associated with AsP in a logistic regression analysis.

	*p* Value	OR	95% CI
Living in nursing home	0.003	4.166	1.641–10.574
Cerebral infarction	0.001	5.002	1.925–13.003
Dementia	0.016	3.360	1.257–8.982
Hypertension	0.029	0.319	0.115–0.888

AsP, aspiration pneumonia; OR, odds ratio; CI, confidence interval.

## Supplementary Materials

The following are available online at https://www.mdpi.com/2308-3417/5/3/42/s1. Table S1: List of Primary Care and General Practice (PCGP) study investigators.

## References

[1] Palacios-Cena D., Hernandez-Barrera V., Lopez-de-Andres A., Fernandez-de-Las-Penas C., Palacios-Cena M., de Miguel-Diez J., Carrasco-Garrido P., Jiménez-García R. (2017). Time trends in incidence and outcomes of hospitalizations for aspiration pneumonia among elderly people in Spain (2003–2013). Eur. J. Intern. Med..

[2] Marik P.E. (2001). Aspiration pneumonitis and aspiration pneumonia. N. Engl. J. Med..

[3] Marrie T.J. (1990). Epidemiology of community-aquired pneumonia in the elderly. Semin. Respir. Infect..

[4] Taylor J.K., Fleming G.B., Singanayagam A., Hill A.T., Chalmers J.D. (2013). Risk factors for aspiration in community-acquired pneumonia: Analysis of a hospitalized UK cohort. Am. J. Med..

[5] Hayashi M., Iwasaki T., Yamazaki Y., Takayasu H., Tateno H., Tazawa S., Kato E., Wakabayashi A., Yamaguchi F., Tsuchiya Y. (2014). Clinical features and outcomes of aspiration pneumonia compared with non-aspiration pneumonia: A retrospective cohort study. J. Infect. Chemother..

[6] Leroy O., Vandenbussche C., Coffinier C., Bosquet C., Georges H., Guery B., Thevenin D., Beaucaire G. (1997). Community-acquired aspiration pneumonia in intensive care units. Epidemiological and prognosis data. Am. J. Respir. Crit. Care Med..

[7] Teramoto S., Fukuchi Y., Sasaki H., Sato K., Sekizawa K., Matsuse T. (2008). Japanese study group on aspiration pulmonary disease. High incidence of aspiration pneumonia in community- and hospital-acquired pneumonia in hospitalized patients: A multicenter, prospective study in Japan. J. Am. Geriatr. Soc..

[8] DiBardino D.M., Wunderink R.G. (2015). Aspiration pneumonia: A review of modern trends. J. Crit. Care.

[9] Janssens J.P., Krause K.H. (2004). Pneumonia in the very old. Lancet Infect. Dis..

[10] Lode H. (1988). Microbiological and clinical aspects of aspiration pneumonia. J. Antimicrob. Chemother..

[11] Kalil A.C., Metersky M.L., Klompas M., Muscedere J., Sweeney D.A., Palmer L.B., Napolitano L.M., O′Grady N.P., Bartlett J.G., Carratalà J. (2016). Management of adults with hospital-acquired and ventilator-associated pneumonia: 2016 Clinical Practice Guidelines by the Infectious Diseases Society of America and the American Thoracic Society. Clin. Infect. Dis..

[12] Japanese Respiratory Society (2017). The JRS Guidelines for the Management of Pneumonia in Adults.

[13] Marrie T.J. (1996). Pneumonia in the elderly. Curr. Opin. Pulm. Med..

[14] Esayag Y., Nikitin I., Bar-Ziv J., Cytter R., Hadas-Halpern I., Zalut T., Yinnon A.M. (2010). Diagnsostic value of chest radiographs in bedridden patients suspected of having pneumonia. Am. J. Med..

[15] Faverio P., Aliberti S., Bellelli G., Suigo G., Lonni S., Pesci A., Restrepo M.I. (2014). The management of community-acquired pneumonia in the elderly. Eur. J. Intern. Med..

[16] Marrie T.J., File T.M. (2016). Bacterial pneumonia in older adults. Clin. Geriatr. Med..

[17] Ishifuji T., Sando E., Kaneko N., Suzuki M., Kilgore P.E., Ariyoshi K., Morimoto K., Hosokawa N., Yaegashi M., Aoshima M. (2017). Recurrent pneumonia among Japanese adults: Disease burden and risk factors. BMC Pulm. Med..

[18] Takeshima T., Kumada M., Mise J., Ishikawa Y., Yoshizawa H., Nakamura T., Okayama M., Kajii E. (2014). Reasons for encounter and diagnoses of new outpatients at a small community hospital in Japan: An observational study. Int. J. Gen. Med..

[19] Loeb M.B. (2005). Pneumonia in nursing homes and long-term care facilities. Semin. Respir. Crit. Care Med..

[20] Jamshed N., Woods C., Desai S., Dhanani S., Taler G. (2011). Pneumonia in the long-term resident. Clin. Geriatr. Med..

[21] Harris P.A., Taylor R., Thielke R., Payne J., Gonzalez N., Conde J.G. (2009). Research electronic data capture (REDCap)–A metadata-driven methodology and workflow process for providing translational research informatics support. J. Biomed. Inform..

[22] Harris P.A., Taylor R., Minor B.L., Elliott V., Fernandez M., O’Neal L., McLeod L., Delacqua G., Delacqua F., Kirby J. (2019). The REDCap consortium: Building an international community of software partners. J. Biomed. Inform..

[23] Marik P.E., Kaplan D. (2003). Aspiration pneumonia and dysphagia in the elderly. Chest.

[24] Kohno S., Imamura Y., Shindo Y., Seki M., Ishida T., Teramoto S., Kadota J., Tomono K., Watanabe A. (2013). Clinical practice guidelines for nursing- and healthcare-associated pneumonia (NHCAP) [complete translation]. Respir. Investig..

[25] American Thoracic Society, Infectious Diseases Society of America (2005). Guidelines for the management of adults with hospital-acquired, ventilator-associated, and healthcare-associated pneumonia. Am. J. Respir. Crit. Care Med..

[26] Kollef M.H., Shorr A., Tabak Y.P., Gupta V., Liu L.Z., Johannes R.S. (2005). Epidemiology and outcomes of health-care-associated pneumonia: Results from a large US database of culture-positive pneumonia. Chest.

[27] Kenzaka T., Kumabe A., Kosami K., Matsuoka Y., Minami K., Ninomiya D., Noda A., Yahata S. (2018). Bacteriological testing and recurrence prevention efforts in the diagnosis and treatment of nursing- and healthcare-associated pneumonia and aspiration pneumonia: A questionnaire survey of hospitals across Japan. Respir. Investig..

[28] Takizawa C., Gemmell E., Kenworthy J., Speyer R. (2016). A systematic review of the prevalence of oropharyngeal dysphagia in stroke, Parkinson’s disease, Alzheimer’s disease, head injury, and pneumonia. Dysphagia.

[29] Nakagawa T., Sekizawa K., Nakajoh K., Tanji H., Arai H., Sasaki H. (2000). Silent cerebral infarction: A potential risk for pneumonia in the elderly. J. Intern. Med..

[30] Manabe T., Teramoto S., Tamiya N., Okochi J., Hizawa N. (2015). Risk factors for aspiration pneumonia in older adults. PLoS ONE.

[31] Diehr P., Williamson J., Burke G.L., Psaty B.M. (2002). The aging and dying processes and the health of older adults. J. Clin. Epidemiol..

[32] Fein A.M. (1999). Pneumonia in the elderly: Overview of diagnostic and therapeutic approaches. Clin. Infect. Dis..

[33] Fleg J.L., O′Connor F., Gerstenblith G., Becker L.C., Clulow J., Schulman S.P., Lakatta E.G. (1995). Impact of age on the cardiovascular response to dynamic upright exercise in healthy men and women. J. Appl. Physiol..

[34] Lakatta E.G. (2003). Arterial and cardiac aging: Major shareholders in cardiovascular disease enterprises: Part III: Cellular and molecular clues to heart and arterial aging. Circulation.

[35] Benjamin E.J., Levy D., Anderson K.M., Wolf P.A., Plehn J.F., Evans J.C., Comai K., Fuller D.L., Sutton M.S. (1992). Determinants of Doppler indexes of left ventricular diastolic function in normal subjects (the Framingham Heart Study). Am. J. Cardiol..

[36] Lim W.S., van der Eerden M.M., Laing R., Boersma W.G., Karalus N., Town G.I., Lewis S.A., Macfarlane J.T. (2003). Defining community acquired pneumonia severity on presentation to hospital: An international derivation and validation study. Thorax.

[37] Satish S., Zhang D.D., Goodwin J.S. (2001). Clinical significance of falling blood pressure among older adults. J. Clin. Epidemiol..

[38] Delgado J., Bowman K., Ble A., Masoli J., Han Y., Henley W., Welsh S., Kuchel G.A., Ferrucci L., Melzer D. (2018). Blood pressure trajectories in the 20 years before death. JAMA Intern. Med..

[39] Kikuchi R., Watabe N., Konno T., Mishina N., Sekizawa K., Sasaki H. (1994). High incidence of silent aspiration in elderly patients with community-acquired pneumonia. Am. J. Respir. Crit. Care Med..

[40] Nakashima T., Maeda K., Tahira K., Taniguchi K., Mori K., Kiyomiya H., Akagi J. (2018). Silent aspiration predicts mortality in older adults with aspiration pneumonia admitted to acute hospitals. Geriatr. Gerontol. Int..

[41] Hase T., Miura Y., Nakagami G., Okamoto S., Sanada H., Sugama J. (2020). Food bolus-forming ability predicts incidence of aspiration pneumonia in nursing home older adults: A prospective observational study. J. Oral Rehabil..

[42] Ikebe K., Matsuda K., Kagawa R., Enoki K., Yoshida M., Maeda Y., Nokubi T. (2011). Association of masticatory performance with age, gender, number of teeth, occlusal force and salivary flow in Japanese older adults: Is ageing a risk factor for masticatory dysfunction?. Arch. Oral Biol..

[43] Hazelwood R.J., Armeson K.E., Hill E.G., Bonilha H.S., Martin-Harris B. (2017). Identification of swallowing tasks from a modified barium swallow study that optimize the detection of physiological impairment. J. Speech Lang. Hear. Res..

[44] Miyashita N., Kawai Y., Tanaka T., Akaike H., Teranishi H., Wakabayashi T., Nakano T., Ouchi K., Okimoto N. (2015). Detection failure rate of chest radiography for the identification of nursing and healthcare-associated pneumonia. J. Infect. Chemother..

