# Supplementary material for: Characteristic Factors of Aspiration Pneumonia to Distinguish from Community-Acquired Pneumonia among Oldest-Old Patients in Primary-Care Settings of Japan
Source: Geriatrics (Basel). 2020 Jul 7;5(3):42. doi: 10.3390/geriatrics5030042 (PMC7555817; doi:10.3390/geriatrics5030042)
Supplement: Supplementary file 1 [file geriatrics-05-00042-s001.pdf]

Supplemental Table 1: List of Primary Care and General Practice (PCGP) Study investigators.

**Co-investigator**	**Affiliation**
Katsuhiro Kusaka	Nanporo Town Hospital, Hokkaido
Yasufumi Matsuoka	Oma Hospital, Aomori
Hideki Munakata	Iwate Prefectural Senmaya Hospital, Iwate
Jun Takahashi	Okitama Public General Hospital, Yamagata
Yoshifumi Uekusa	Kita-ibaraki City Hospital, Ibaraki
Maki Kumada	Nasu-minami Hospital, Tochigi
Hidenori Kamiyama	Chichibu City Hospital, Saitama
Daisuke Inoue	Okutama Hospital, Tokyo
Shigeru Koyama	Tokyo Metropolitan Hiroo Hospital, Tokyo
Hideo Hirose	Wara Public Clinic. Gifu
Hiroyaki Kawashiri	Takayama City Takane Clinic, Gifu
Susumu Nakayama	Minami-nara General Medical Center, Nara
Shigehisa Sakurai	Chizu Hospital, Tottori
Jun Watanabe	Saji Clinic, Tottori
Eiichi Kakehi	Tottori City Hospital, Tottori
Junji Mashino	Shimane Prefectural Central Hospital, Shimane
Yuji Okazaki	Aki-ohta Hospital, Hiroshima
Atsushi Takayama	Mishima Clinic, Yamaguchi
Nobuki Nanki	Sanuki City Hospital, Kagawa
Kentaro Kasahara	Aozora Gastrointestinal Clinic, Saga
Hideto Sonoda	Imari-Arita Kyoritsu Hospital, Saga
Hiroyuki Teraura, Kazuhiko Kotani, Masami Matsumura	Organizing office; Jichi Medical University, Tochigi
